# 

**Published:** 1883-06

**Authors:** 


### CONTENTS:

PAGE.

ORIGINAL COMMUNICATIONS:

I. What is Disease? By W. Godfrey Dyas, F.R.C.S. .... 561

II. Cough of Remote Origin. By W. E. Casselberry, M.D. .... 570

III. The Pulsation of Veins .... 577

SOCIETY REPORTS:

IV. Illinois State Board of Health—Quarterly Meeting .... 581

V. Chicago Medical Society .... 602

VI. Michigan State Board of Health .... 607

VII. Chicago Pathological Society .... 611

DOMESTIC CORRESPONDENCE:

VIII. Letter from Elgin .... 614

PAGE.

REVIEWS AND BOOK NOTICES .... 615

EDITORIAL:

Medical Examinations by State Boards 620

ORIGINAL TRANSLATIONS .... 626

SELECTIONS:

Remarks on 177 Operations for Entropium and Trichiasis. By F. C. Hotz, M.D. .... 640

Clinical Remarks on the Functional Vomiting of Hysteria. By J. S. Bristowe, M.D. .... 648

Locomotor Ataxy .... 661

ITEMS .... 580, 606, 613, 619, 622 663

NOTICE TO CORRESPONDENTS .... *Advts*

